# Evaluation of the Anticonvulsant and Anxiolytic Potentials of Methyl Jasmonate in Mice

**DOI:** 10.3797/scipharm.1310-22

**Published:** 2014-03-24

**Authors:** Olajide S Annafi, Solomon Umukoro, Anthony T Eduviere

**Affiliations:** ^1^ Department of Pharmacology and Therapeutics, University of Ibadan, Ibadan, Nigeria.

**Keywords:** Methyl jasmonate, Picrotoxin, Pentylenetetrazole, Strychnine, Convulsions, Anxiolytic

## Abstract

Methyl jasmonate (MJ) is one of the most well-studied plant stress hormones belonging to the jasmonate family. Previous studies have shown that MJ potentiated pentobarbitone sleeping time and enhanced GABA-mediated inhibitory neurotransmission, suggesting potential benefits in disorders associated with hyperactivity of the brain. This study was carried out to evaluate whether MJ has anticonvulsant and anxiolytic properties in mice. The anticonvulsant effect was assessed based on the prevention of tonic-clonic seizures induced by chemoconvulsant agents in mice. The anxiolytic property was evaluated utilizing the elevated plus maze (EPM) and light/dark transition paradigms. The effect of MJ on spontaneous locomotor activity (SMA) was also assessed. Mice received intraperitoneal (i.p.) injections of MJ 30 min before the tests were carried out and diazepam (2 mg/kg, i.p.) was used as the reference drug. MJ (50–400 mg/kg) did not protect the mice against tonic-clonic convulsions induced by picrotoxin (10 mg/kg, i.p.) or strychnine (3 mg/kg, i.p.). However, MJ (100, 200, and 400 mg/kg) offered 20, 60, and 100% protection against pentylenetetrazole (100 mg/kg, i.p.)-induced convulsions. In a similar manner to diazepam (2 mg/kg), MJ (400 mg/kg) produced a marked sedative effect as shown by decreases in the number of lines crossed and the duration of ambulation in the open field test. In contrast to diazepam (2 mg/kg), MJ (5–50 mg/kg) did not show anxiolytic effects in the EPM and light/dark transition paradigms. These findings suggest that methyl jasmonate at high doses possessed anticonvulsant properties in the pentylenetetrazole animal model of epilepsy, but did not produce anxiolytic activity in mice.

## Introduction

Convulsive seizures and anxiety are central nervous system disorders associated with an imbalance between excitatory and inhibitory mechanisms of the brain and impose untold suffering on patients [1 – 3]. Convulsion manifests itself in forms of recurrent seizures, whereas anxiety is related to sustained anxious feelings in response to a perceived life threatening situation [1, 2, 4]. Anxiety shares common neurochemical pathways with convulsions such as reduced GABAergic and increased glutaminergic neurotransmissions [2, 4]. Although large proportions of people suffer from these ailments worldwide, the development of new pharmacological interventions has lagged behind in comparison with many other therapeutic counterparts [4]. The standard anticonvulsant and anxiolytic drugs have a limited spectrum of activities with adverse effects that have limited their clinical usefulness and compromised patients’ compliance [2, 4]. Thus, there is need to search for newer agents with better clinical profiles for the treatment of convulsions and relief of anxiety symptoms.

Methyl jasmonate (MJ) is a cyclopentanoic compound secreted by plants in response to external stressors [5]. MJ was first isolated from the essential oil of Jasminum grandiflorum, but is now obtained through chemical synthesis [6]. Previous investigations have shown that MJ has anticancer, antinociceptive, antiaggressive, and antidepressant properties [5, 7, 8]. In addition, Hossain et al. [9] reported that MJ potentiated pentobarbitone sleeping time and enhanced GABA-mediated inhibitory neurotransmission, suggesting the potential benefits in disorders associated with hyperexcitability of the brain such as convulsions and anxiety states. Thus, this present study was carried out to evaluate whether MJ exhibits anticonvulsant and anxiolytic activities in mice.

## Experimental

### Laboratory Animals

Albino Swiss mice of either sex (18–22 g) were obtained from the Animal Centre, University of Ibadan, Nigeria and were kept in plastic cages at room temperature. They were fed with balanced rodent pellet diet and water ad libitum and were acclimatized for one week before using them for experiments. The procedures used in the study were in compliance with the ethical guidelines of the University of Ibadan and National Institutes of Health guide for the Care and Use of Laboratory Animals for experimental investigations.

### Drugs and Treatment

The following drugs were used: picrotoxin (Sigma-Aldrich, St. Louis, USA), pentylenetetrazole, PTZ (Sigma-Aldrich, St. Louis, USA), strychnine (Sigma, USA), Diazepam, DPZ (Sigma,USA), and methyl jasmonate, MJ (Sigma-Aldrich Chemie GmbH, Steinheim, Germany). Picrotoxin, pentylenetetrazole, and strychnine were dissolved in distilled water. One hundred milligrams of MJ were dissolved in 1 ml of ethanol (95%) to give 100 mg/ml stock solution. This stock solution was further diluted with distilled water to obtain the different concentrations used in the study. The final concentration of ethanol in each solution used for the study did not exceed 1%.

### Effect of Methyl Jasmonate on Picrotoxin-Induced Convulsions

The effect of MJ on picrotoxin-induced convulsions was evaluated according to the method described by Loscher [10]. Mice (n = 6) were treated with intraperitoneal (i.p.) injections of MJ (50–400 mg/kg), DPZ (2 mg/kg), or a vehicle (10 ml/kg of 1.0% ethanol) 30 min prior to the induction of convulsions with i.p, injections of picrotoxin (10 mg/kg). Thereafter, the animals were placed individually in a transparent chamber and the latency to tonic-clonic seizures as well as the mortality rate was assessed for the period of 30 min after the administration of picrotoxin [10].

### Effect of Methyl Jasmonate on Convulsions Induced by Pentylenetetrazole

The PTZ animal model of epilepsy was further used to evaluate the anticonvulsant property of MJ following the procedure previously described by [11]. Mice were treated with i.p. injections of MJ (50–400 mg/kg), DPZ (2 mg/kg), or a vehicle (10 ml/kg of 1.0% ethanol) 30 min before the induction of seizures with i.p. injections of PTZ (100 mg/kg). The animals were then observed for the appearance of tonic-clonic seizures and death as earlier described [11].

### Effect of Methyl Jasmonate on Convulsions Induced by Strychnine

The effect of MJ on seizures induced by strychnine was also evaluated in this study as described above. Mice were treated with i.p. injections of MJ (50–400 mg/kg), DPZ (2 mg/kg), or a vehicle (10 ml/kg of 1.0% ethanol) 30 min prior to the induction of convulsions with strychnine (3 mg/kg, i.p.) and then monitored for the appearance of tonic-clonic seizures and death.

### Performance of Methyl Jasmonate in the Elevated Plus-Maze Test

The EPM animal model of anxiety was used to evaluate the anxiolytic property of MJ according to the procedure previously described [12]. Mice were randomly distributed into six treatment groups (n = 5). The first four groups received MJ (5, 10, 20, and 50 mg/kg), whereas the fifth group was treated with DPZ (2 mg/kg). The last group received a vehicle (10 ml/kg of 1.0% ethanol), which served as a negative control. Thirty minutes later, each mouse was placed at the centre of the maze, facing an open arm and allowed to explore it for 5 min. The number of entries and time spent (s) in each arm of the EPM were recorded for the period of 5 min.

### Performance of Methyl Jasmonate in the Light/Dark Transition Test

The light/dark transition test was further employed to assess the anxiolytic property of MJ according to the method of [13]. Mice (five per group) were treated with MJ (5, 10, 20, and 50 mg/kg, i.p.), DPZ (2 mg/kg, i.p.), or a vehicle (10 ml/kg of 1.0% of ethanol, i.p.). Thirty minutes after treatment, each mouse was placed in the illuminated compartment of the box. Thereafter, the number of entries and time spent in the light and dark compartments of the box were measured for the 5 min session [14].

### Effect of Methyl Jasmonate on Spontaneous Locomotor Activity in the Open Field Test

The open field test as a measure of spontaneous locomotor activity was utilized to evaluate the sedative property of MJ following the procedure described by Bruhwyler et al. [14]. Mice (five per group) received i.p. injections of MJ (5–400 mg/kg.), DPZ (2 mg/kg, i.p.), or a vehicle (10 ml/kg of 1.0 % of ethanol) and were placed individually at the centre of the box 30 min later. Thereafter, the number of line crosses and duration of ambulation were measured for the 5 min period.

### Statistical Analysis

The data were expressed as the mean ± S.E.M. The data were analyzed with Graph Pad Prism software version 4.03. Statistical analysis of the data was done by One-way ANOVA, followed by Newman-Keuls multiple comparison test. P-values less than 0.05 were considered statistically significant.

## Results and Discussion

### Effect of Methyl Jasmonate on Picrotoxin-Induced Convulsions

Mice treated with the vehicle exhibited tonic-clonic convulsions with a latency of 5.56±0.13 seconds after the i.p. injection of picrotoxin (10 mg/kg, i.p.) and all of the animals died after seizures. One-way ANOVA revealed that there were significant differences between the treatment groups: latency to convulsions [F_(5, 24)_ = 19.16, *P* = 0.0097] and latency to death [F_(5, 24)_ = 20.73, *P* <0.0001]. Post-hoc analysis by the Newman-Keuls multiple comparison test showed that MJ (200–400 mg/kg) as well as DPZ (2 m/kg) delayed the latency to the tonic-clonic components of picrotoxin-induced seizures in mice (Table 1). In addition, MJ (200–400 mg/kg) as well as DPZ only delayed the latency to death, as all of the animals eventually died (Table 1).

**Tab. 1.** Effect of methyl jasmonate on picrotoxin-induced convulsions

Treatment Group	Dose (mg/kg)	Latency to Convulsion (min)	Latency to Death (min)	Convulsion (%)/Death (%)
Control	–	5.56±0.13	10.6±0.30	100/100
MJ	50	6.04±0.55	11.2±0.26	100/100
MJ	100	6.11±0.41	11.3±0.31	100/100
MJ	200	6.26±0.29	11.4±0.51	100/100
MJ	400	9.56±0.64*	19.6±0.55*	100/100
DZP	2	11.20±0.85*	17.8±0.56	100/100

Values represent the Mean ± S.E.M for 5 animals per group.

*P<0.05 compared to control (ANOVA followed by the Newman-Keuls multiple comparison Test. Latency to convulsion [F (_5, 24_) = 19.16, P = 0.0097] and latency to death [F_(5, 24)_ = 20.73, P<0.0001].

### Effect of Methyl Jasmonate on Pentylenetetrazole-Induced Convulsions

The effects of MJ (50–400 mg/kg, i.p) on convulsions induced by PTZ (100 mg/kg, i.p) in mice are shown in Table 2. One-way ANOVA showed that there were significant differences between the treatment groups: latency to convulsions [F_(5, 24)_ = 31.03, *P*<0.0001] and latency to death [F_(5, 24)_ = 180.1, *P*<0.0001]. Post-hoc test analysis by the Newman-Keuls multiple comparison test showed that MJ (100–400 mg/kg) increased the latency to the tonic-clonic components of PTZ-induced seizures in mice (Table 2). At these doses, MJ reduced the mortality rate, whereas the death rate was 100% in the control group (Table 2). The reference drug, diazepam (2 mg/kg) offered 100% protection against convulsions, as none of the animals exhibited tonic-clonic seizures induced by PTZ in mice (Table 2).

**Tab. 2.** Effect of methyl jasmonate on pentylenetetrazole-induced convulsions

Treatment	Dose (mg/kg)	Latency to Convulsion (min)	Latency to Death (min)	Convulsion (%)/Death(%)
Control	–	0.89±0.14	1.3±0.04	100/100
MJ	50	1.06±0.13	1.6±0.04	100/100
MJ	100	1.36±0.17*	2.7±0.08*	80/80
MJ	200	1.75±0.19*	3.2±0.07*	60/40
MJ	400	–	–	0/0
DZP	2	–	–	0/0

Values represent the Mean ± S.E.M for 5 animals per group.

Values represent the Mean ± S.E.M for 5 animals per group.

*P<0.05 compared to control (ANOVA followed by the Newman-Keuls multiple comparison test). Latency to Convulsion [F_(5, 24)_ = 31.03, P<0.0001] and latency to death [F_(5, 24)_ = 180.1, P<0.0001].

### Effect of Methyl Jasmonate on Strychnine-Induced Convulsions

One-way ANOVA showed that there were no significant differences between the treatment groups: latency to convulsions [F_(5, 24)_ = 3.921, *P* = 0.0097] and latency to death [F_(5, 24)_ = 130.9, *P*<0.0001]. Post-hoc analysis by the Newman-Keuls multiple comparison test revealed that MJ (50–400 mg/kg) did not significantly (p > 0.05) prolong the latency to convulsions produced by strychnine in mice (Table 3). In addition, it did not offer any protection against convulsions, as all of the animals experienced tonic-clonic seizures. As shown in Table 3, a similar pattern of effects were observed in the group given DPZ (2 mg/kg, i.p.).

**Tab. 3.** Effect of methyl jasmonate on strychnine-induced convulsions in mice

Treatment Group	Dose (mg/kg)	Latency to Convulsion (min)	Latency to Death (min)	Convulsion (%)/Death %)
Control	–	2.36±0.29	2.4±0.02	100/100
MJ	50	2.38±0.17	2.5±0.02	100/100
MJ	100	2.41±0.26	2.6±0.02	100/100
MJ	200	2.44±0.28	2.6±0.04	100/100
MJ	400	3.57±0.58	3.7 ±0.39*	100/100
DZP	2	3.18±0.19	3.2±0.06	100/100

Values represent the Mean ± S.E.M for 5 animals per group.

*P<0.05 compared to control (ANOVA followed by the Newman-Keuls multiple comparison test). Latency to convulsion [F_(5, 24)_ = 3.921, P = 0.0097] and latency to death [F_(5, 24)_ = 130.9, P<0.0001].

### Performance of Methyl Jasmonate in Elevated Plus-Maze Paradigm

**Fig. 1.**
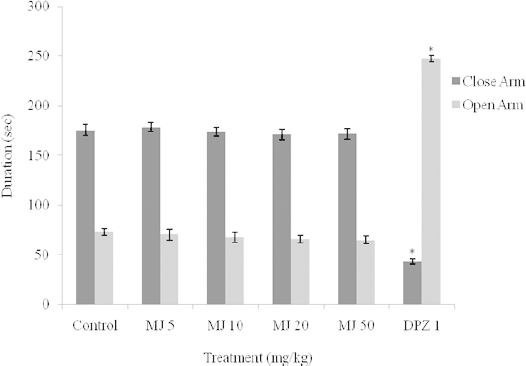
Effect of methyl jasmonate on time spent in closed and open arms in the elevated plus maze. Values represent the Mean ± S.E.M for 5 animals per group. *P<0.05 compared to control (ANOVA followed by the Newman-Keuls multiple comparison test). Time spent in the closed arm [F_(5, 24)_ = 122.6, P <0.0001]; Time spent in the open arm [F_(5, 24)_ = 295.4, P<0.0001].

**Fig. 2.**
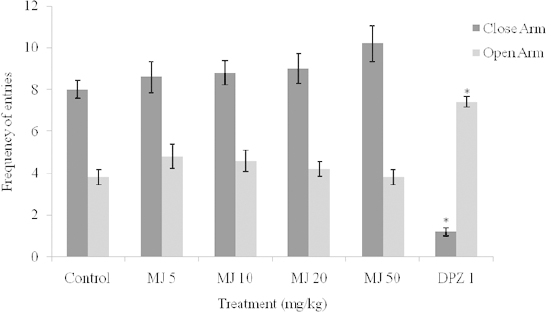
Effect of methyl jasmonate on the frequency of entries into the closed and open arms in the elevated plus maze. Values represent the Mean ± S.E.M for 5 animals per group. *P<0.05 compared to control (ANOVA followed by the Newman-Keuls multiple comparison test). Number of opened arm entries [F_(5, 24)_ = 10.17, P<0.0001] and number of closed arm entries [F_(5, 24)_ = 26.36, P < 0.0001].

The effect of MJ on the explorative behaviors of mice in the EPM test, as assessed by the number of arm entries and time spent in the arms, are shown in figures 1 and 2. One-way ANOVA revealed that there were significant differences between the treatment groups: the number of open arm entries [F_(5, 24)_ = 10.17, *P*<0.0001] and number of closed arm entries [F_(5, 24)_ = 26.36, *P*<0.0001]; time spent in the open arm [F_(5, 24)_ = 295.4, *P*<0.0001] and time spent in the closed arm [F_(5, 24)_ = 122.6, *P*<0.0001]. Post-hoc analysis by the Newman-Keuls multiple comparison test revealed that MJ (5, 10, 20, 50 mg/kg, i.p.) did not significantly (p > 0.05) increase the number of open arm entries and duration of time spent in the open arm as compared with the vehicle, which suggests absence of anxiolytic property. In contrast, DPZ (2 mg/kg, i.p.) showed anxiolytic effects as it significantly (p < 0.05) modified all of the components of the EPM paradigm (Fig. 1 and 2).

### Performance of Methyl Jasmonate in the Light/Dark Transition Test

**Fig. 3.**
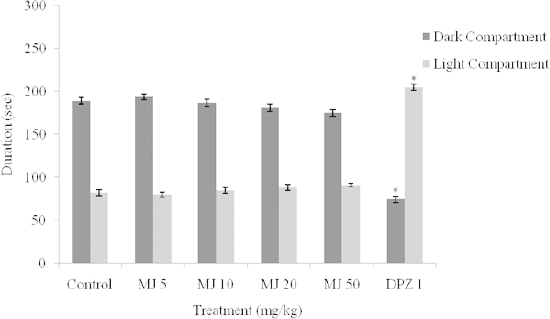
Effect of methyl jasmonate on the time spent in the dark and light compartments in the light and dark box. Values represent the Mean ± S.E.M for 5 animals per group. *P<0.05 compared to control (ANOVA followed by the Newman-Keuls multiple comparison test). Time spent in the light compartment [F_(5, 24)_ = 231.2, P<0.0001] and time spent in the dark compartment [F_(5, 24)_ = 133.8, P<0.0001].

The effect of MJ on the performance of mice in the light/dark transition test, as measured by the amount of time spent in the light and dark compartments, are shown in Figure 3. One-way ANOVA showed that there were significant differences between the treatment groups: the time spent in the light compartment [F_(5, 24)_ = 231.2, *P* < 0.0001] and time spent in the dark compartment [F_(5, 24)_ = 133.8, *P*<0.0001]. Post-hoc analysis by the Newman-Keuls multiple comparison test showed that MJ (5, 10, 20, 50 mg/kg) did not significantly (p > 0.05) increase the amount of time spent in the light compartment when compared with the vehicle, which further suggests absence of anxiolytic property in mice (Fig. 3).

However, DPZ (2 mg/kg) significantly (p < 0.05) increased the duration of time spent in the light compartment in comparison with the vehicle, indicating anxiolytic effects (Fig. 3).

### Sedative Effect of Methyl Jasmonate

The effect of MJ on SMA as assessed by the number of line crosses and duration of ambulation in the open field test are presented in Table 4. One-way ANOVA revealed that there were significant differences between the treatment groups: the number of line of crosses [F(_9, 40_) = 46.59, *P*<0.0001] and duration of ambulation [F(_9, 40_) = 43.63, *P*<0.0001]. Post-hoc analysis by the Newman-Keuls multiple comparison test revealed that MJ (400 mg/kg) produced marked sedation as shown by the reduced number of line crosses and duration of ambulation in mice (Table 4). However, at lower doses, it did not significantly change the SMA when compared with the control value (Table 4).

**Tab. 4.** Effect of methyl jasmonate on spontaneous motor activity in mice

Treatment Group	Dose (mg/kg)	Number of line crossed	Duration of ambulation (s)
Control	–	70.60±4.23	80.40±5.20
MJ	5	71.40±3.87	81.80±5.09
MJ	10	73.80±4.12	83.40±4.77
MJ	20	75.20±2.75	85.00±3.87
MJ	50	77.80±4.08	86.60±6.40
MJ	200	65.60±3.59	66.60±4.90*
MJ	400	2.20±0.37*	2.60±0.25*
DZP	2	33.20±2.42*	35.00±2.72*

Values represent the Mean ± S.E.M for 5 animals per group.

^*^P<0.05 compared to control (ANOVA followed by the Newman-Keuls multiple comparison test). Number of line crosses [F_(8,36)_ = 53.41, P<0.0001] and duration of ambulation [F_(8, 36)_ = 46.98, P<0.0001].

## Discussion

The results of this study showed that MJ given intraperitoneally offered significant protection against tonic-clonic convulsions induced by PTZ in mice. At the dose of 400 mg/kg, it blocks the action of PTZ, as none of the animals exhibited toinc-clonic convulsions. However, MJ could only delay the onset of seizures, as all of the animals experienced convulsions induced by picrotoxin. In a similar manner to diazepam, the reference drug, MJ did not modify the different components of strychnine-induced seizures. In the open field test, MJ at high doses suppressed spontaneous locomotor activity as shown by decreases in the duration of ambulation and the number of lines crossed. However, in contrast to diazepam, MJ did not exhibit anxiolytic activity at doses used in this study in the EPM and light/dark animal paradigms of anxiety.

Picrotoxin, pentylenetetrazole, and strychnine are widely used to induce convulsions in experimental animals [1, 4, 15]. However, the PTZ animal model has been shown to more closely reflect clonic convulsive seizures seen in humans and is known to be sensitive to the blocking action of anticonvlsant drugs [1, 4]. Pentylenetetrazole induces convulsions by antagonizing gamma aminobutyric acid (GABA) in a competitive manner [15]. The antagonism of the postsynaptic GABA receptor leads to excessive neuronal excitation that terminates in convulsions and death in laboratory animals [1, 15, 16]. Picrotoxin is a non-competitive antagonist of the GABA receptor chloride ion complex, whereas strychnine causes convulsions through the competitive antagonism of glycine at glycinergic receptors in the spinal cord [16 – 19]. The major indicator of the anticonvulsant property of a novel compound is based on the prevention of tonic-clonic convulsions especially in the PTZ animal model of epilepsy [1, 4]. Thus, the inhibitory effect shown by MJ against PTZ-induced seizure episodes suggests anticonvulsant activity. In addition, MJ significantly delayed the onset of seizures produced by both PTZ and picrotoxin, which further suggest a beneficial role in retarding the spread of seizures in epileptic brains [20]. Conversely, the failures of MJ and diazepam to modify the convulsant action of strychnine clearly show a lack of effect on the glycinergic system. It is worthy to note that DPZ mediates its anticonvulsant activity through the enhancement of GABA_A_-mediated inhibitory neuro-transmission [1, 10, 21]. This may perhaps explain its efficacy in convulsions induced by PTZ [22].

Generally, drugs which enhanced GABA currents are known to exhibit anticonvulsant activity against PTZ, but might be weak or ineffective against picrotoxin-induced convulsions [4, 21, 23]. The apparent insensitivity of picrotoxin to MJ may be related to the site of its action at the chloride ion gate, which is not readily accessible to most anticonvulsant drugs [18, 23]. Thus, the inability to gain access to the closed chloride ion gate in order to reopen it might explain the failure of MJ to prevent seizures due to picrotoxin. It is our opinion that the enhancement of GABA currents previously reported for MJ [9] might perhaps account for its ability to modify some of the components of convulsions induced by PTZ and picrotoxin in mice. However, further studies using experimental models such as kindling, are necessary to determine if MJ is able to modify the epileptogenesis process.

The EPM and light/dark transition tests are animal models commonly used for elucidating the neurobiological mechanisms of anxiety and for detecting drugs with anxiolytic properties [24]. The behavioral alteration seen in EPM is based on the natural aversion of rodents for open environments. Thus, mice taken from the home cage naturally show a pattern of behavior characterized by open arm avoidance with consistent preference for closed arms [4, 24]. This behavioral trait is suppressed by anxiolytics and enhanced by anxiogenics [4, 24]. Thus, the measures of anxiety in the EPM paradigm are based on the alterations in the number of open arm entries and the total time spent in the open arm [24, 25]. In this model, MJ at the tested doses did not significantly alter the number of open arm entries and time spent in the open arm, suggesting absence of anxiolytic property. Similarly, the natural tendency for rodents is to avoid the light compartment and show preference for the dark region of the light/dark apparatus [26]. MJ also failed to significantly modify the behavioral performance of the animals in the light/dark transition test, which further suggests absence of anxiolytic property. However, gross sedating effect was observed at high doses of MJ in the open field test. Traditionally, the open field test has served as the animal paradigm for assessing pharmacological agents with CNS stimulants or depressant activity [14, 27]. In this test, MJ suppressed SMA at high doses, which suggests sedative or central depressant effect.

## Conclusion

This study suggests that methyl jasmonate possessed anticonvulsant properties in the PTZ animal model of epilepsy, but did not demonstrate anxiolytic activity in mice.
